# *McSAS*: software for the retrieval of model parameter distributions from scattering patterns

**DOI:** 10.1107/S1600576715007347

**Published:** 2015-05-22

**Authors:** I. Bressler, B. R. Pauw, A. F. Thünemann

**Affiliations:** a BAM Federal Institute for Materials Research and Testing, 12205 Berlin, Germany; b National Institute for Materials Science, 305-0047 Tsukuba, Japan

**Keywords:** small-angle scattering, Monte Carlo, data analysis software, disperse samples

## Abstract

A user-friendly open-source Monte Carlo regression package (*McSAS*) is presented, which structures the analysis of small-angle scattering (SAS) using uncorrelated shape-similar particles (or scattering contributions). The underdetermined problem is solvable, provided that sufficient external information is available. Based on this, the user picks a scatterer contribution model (or ‘shape’) from a comprehensive library and defines variation intervals of its model parameters. A multitude of scattering contribution models are included, including prolate and oblate nanoparticles, core–shell objects, several polymer models, and a model for densely packed spheres. Most importantly, the form-free Monte Carlo nature of *McSAS* means it is not necessary to provide further restrictions on the mathematical form of the parameter distribution; without prior knowledge, *McSAS* is able to extract complex multimodal or odd-shaped parameter distributions from SAS data. When provided with data on an absolute scale with reasonable uncertainty estimates, the software outputs model parameter distributions in absolute volume fraction, and provides the modes of the distribution (*e.g.* mean, variance *etc.*). In addition to facilitating the evaluation of (series of) SAS curves, *McSAS* also helps in assessing the significance of the results through the addition of uncertainty estimates to the result. The *McSAS* software can be integrated as part of an automated reduction and analysis procedure in laboratory instruments or at synchrotron beamlines.

## Abstract 2

A user-friendly open-source Monte Carlo regression package (*McSAS*) is presented, which aids in the analysis of scattering patterns from uncorrelated, shape-similar scatterers. The Monte Carlo nature necessitates an assumption on the elementary shape of the scatterer, but can resolve the shape-defining parameter distributions without restrictions on the mathematical form of the distribution.

## 1. Introduction   

Quantification of nanoscale structures is set to become a requirement in industrial preparation of materials (EU, 2011 ▶). Therefore, a toolset is desired to obtain quantitative morphological parameter distributions of (size-)disperse nanoparticle mixtures with minimal effort, high flexibility, accuracy and high reliability.

The most commonly used technique for nanostructural quantification is transmission electron microscopy (TEM). TEM is essential in determining the overall morphology of the nanostructural features and can often be used to coarsely quantify their parameters. Obtaining a statistically representative quantification of the nanostructure, however, is reliant on the probing of large numbers of objects. To improve its representation of the bulk of the sample, it should preferably be performed through sampling from multiple locations throughout a bulk-scale sample (Klein *et al.*, 2011 ▶; Meli *et al.*, 2012 ▶). As TEM has remained largely resilient to automation efforts, this continues to be a tedious and labour-intensive task. It is, therefore, beneficial to combine the localized resolving power of microscopy with another technique more suited for bulk-scale nanostructural quantification such as small-angle scattering (SAS) (ISO, 2014 ▶; Pauw, 2013 ▶).

SAS offers one reliable route to bulk quantification of materials: it can characterize the nanostructure of large amounts of material with a minimum of tedium, easily extracting size distributions and volume fractions (for example). Practically, however, one of the biggest stumbling blocks in its application has been the data correction and analysis. Although the discussion of data corrections is beyond the scope of this work [see Jacques *et al.* (2012 ▶), Pauw (2013 ▶) and Kieffer & Karkoulis (2013 ▶) for such discussions], it has to be stressed that correct analysis of data is reliant on the quality thereof. There can be no good results without proper data which, in turn, cannot be considered complete without reasonable uncertainty estimates on the data values. Prior work has shown that reasonable data uncertainty may be estimated as the maximum of the values given (*a*) by the standard error of the mean (obtained during the averaging or binning procedure), (*b*) by propagating photon counting uncertainties through the data corrections and (*c*) by limiting the data uncertainty to be no less than 1% of the data value (Pauw, 2013 ▶; Rosalie & Pauw, 2014 ▶; Schnepp *et al.*, 2013 ▶).

After suitably corrected SAS data have been obtained, analysis thereof can be performed through a classical approach: using a least-squares optimization to match the measured data to a model scattering pattern, defined by a handful of model parameters (Pedersen, 1997 ▶). However, the assumptions made in such model functions [on both the scatterer shape and the mathematical form of the parameter distribution(s)] are often insufficiently flexible to describe the morphology of many samples. Good agreement between the model function and the measured data will then not be achieved, in particular for samples where the actual dispersity does not adhere to the inherently assumed model parameter distribution form (such as lognormal, Gaussian or Schultz–Zimm), or where such a distribution form is not known or can not be assumed *a priori*.

Modern analysis methods are available for this class of samples (*e.g.* size-disperse) which allow for the retrieval of model parameter distributions without assumptions on the form of the distribution. While the general shape of the scatterer still has to be defined in order to arrive at a unique solution [see, for example, Rosalie & Pauw (2014 ▶)], the methods are no longer restricted to a limited set of model parameter distribution forms. Such modern methods include Titchmarsh (Fedorova & Schmidt, 1978 ▶; Botet & Cabane, 2012 ▶) or indirect Fourier transforms, based either on smoothness criteria (Glatter, 1977 ▶; Svergun, 1991 ▶), maximum entropy optimization (Hansen & Pedersen, 1991 ▶) or Bayesian hyperparameter estimation (Hansen, 2000 ▶). While these carry a certain mathematical elegance, they can be challenging to implement, understand and apply. This mathematical obscurity furthermore hinders thorough understanding of the failure modes, which can lead to crucial errors in their application.

Recently, a conceptually straightforward Monte Carlo-based method was presented for determining model parameter distributions from small-angle scattering patterns (Pauw, Pedersen *et al.*, 2013 ▶), which has since been applied to explore the size distributions of a variety of samples including metal alloys (Rosalie & Pauw, 2014 ▶), novel oxygen reduction reaction catalysts (Schnepp *et al.*, 2013 ▶), polymer fibres (Pauw, Ohnuma *et al.*, 2013 ▶), plasmoids (Meir *et al.*, 2013 ▶), iron oxide nanoparticles (Lak *et al.*, 2014 ▶) and quantum dots (Abécassis *et al.*, 2015 ▶). While these results have been encouraging, the lack of user friendliness of the method has hindered its adoption by a broader audience.

Through a multinational collaborative effort spanning several years, a drastic improvement on the software usability was effected. This was mostly accomplished through a comprehensive rewrite of the implementation following modern coding standards and conventions, and the addition of a graphical user interface. After a brief recapitulation of the method concept, the software capabilities and interface are detailed, and application examples are given.

## 2. *McSAS* fitting procedure   

### 2.1. Core concept   

The *McSAS* method is a Monte Carlo rejection sampling approach for retrieving model parameter distributions, such as size distributions, from scattering patterns (Pauw, Pedersen *et al.*, 2013 ▶). Central to the method lies a set of independent non-interacting contributions, each of which is an instance of the elementary scatterer model chosen by the user. Depending on the chosen model, one or more parameters can be selected for Monte Carlo optimization (hereafter, referred to as ‘fitting parameter’).

If the measured data are provided in absolute units, calculations can be performed resulting in absolute volume fractions. In order for this to work, information on the scattering length densities of the phases within the sample needs to be provided. For the included two-phase models, only the differences of the scattering length density of the scatterer and that of the matrix are necessary. These scattering length densities are readily obtained from a variety of online tools (Brown & Kienzle, 2015 ▶).

**Figure 1:**
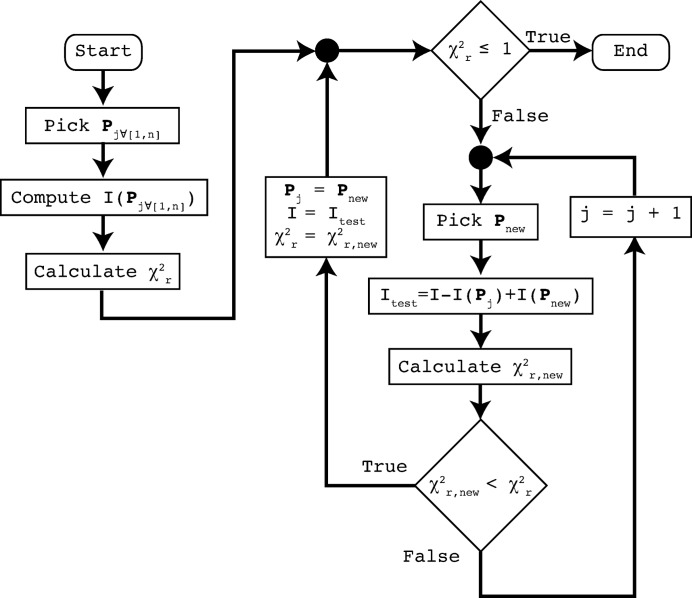
The main process of the *McSAS* software for parameter optimization. In each cycle, an attempt is made to replace one of the model contributions in order to improve the agreement between model and measured data.

The optimization procedure (shown in Fig. 1 ▶) progresses through replacement of contributions in the set. At the end of the optimization procedure, the spread of fitting parameter values of the contributions in this set defines the final parameter distribution.

### 2.2. Optimization procedure   

The method starts from a set of non-interacting scatterers of predefined shape (*e.g.* spheres, rods, ellipsoids) but with random values chosen for the fitting parameter(s) of each contribution in the set. The total model scattering pattern is given by the (weighted^1^) sum of the scattering patterns of each scatterer in the set.

A figure of merit (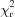) is calculated from the model based on the distance between the model and measured data, weighted by the measured data uncertainty estimates (Pedersen, 1997 ▶). In order to obtain this figure of merit, the model intensity is matched to the measured data set through scaling and addition of an optional flat background contribution. The scaling and background parameters are obtained by least-squares minimization of 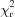. The figure of merit thus indicates when the model describes the data on average to within the data uncertainty (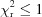). Thereby, a suitable cutoff criterion for optimization is provided, which generally prevents over-fitting of the data and allows for the estimation of uncertainties on the resultant distribution.

Each iteration of the Monte Carlo (MC) procedure consists of replacing one of the scattering objects in the set by another object of the same basic shape but with different, randomly chosen values for its fitting parameter(s). This replacement is accepted if it reduces 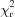, *i.e.* if the agreement of the resulting MC scattering pattern with the measured pattern is improved. These iterations continue until the convergence criterion of 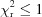 is reached (the convergence criterion value can be adjusted by the user to support data with over- or underestimated uncertainties). After completion, the model parameter distribution is determined through grouping (binning) of the fitting parameter values in the set.

In addition to this, a ‘minimum observability limit’ is determined for each contribution in the set, which specifies the minimum volume fraction of scatterers required to make a *measurable* contribution to the scattering pattern (*i.e.* a contribution exceeding the measurement uncertainty). More specifically, a minimum observability limit 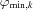 (in units of volume fraction) can be defined for any method where the total model intensity comprises a set of quantized components, whose partial contributions are 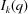 for a given component volume fraction 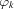, and where the measurement data uncertainty 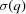 is available:
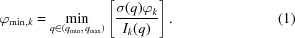
Its derivation and use is further explored elsewhere (Pauw, Pedersen *et al.*, 2013 ▶).

Finally, the uncertainty on the resultant parameter distribution is determined through analysis of the sample standard deviation of a multitude of independent MC solutions. These uncertainty estimates and the observability limits are key values in the application of the method. They provide information to distinguish between numerical noise and size distribution components which are shown by the data and, moreover, allow for the assessment of the statistical significance of differences in resultant size distributions. The accuracy of such uncertainty estimates and observability limits in the *McSAS* result are, however, directly reliant on the provision of reasonable uncertainty estimates on the measured data.

With this procedure, *McSAS* is able to retrieve any form-free size distribution provided a basic scatterer shape is given. A test of the retrievability of a wide range of unimodal and multimodal size distributions has been demonstrated for a large variety of simulated size distributions in the supplementary information given by Pauw, Pedersen *et al.* (2013 ▶). A comparison between size distributions of precipitates in alloys is also available, obtained from electron microscopy and *McSAS* analysis of small-angle X-ray scattering (SAXS) data (Rosalie & Pauw, 2014 ▶).

### 2.3. MC method benefits and drawbacks   

#### 2.3.1. Features   

*McSAS* has proven to be remarkably useful owing to its ability to work in absolute units and the wide variety of available models. These models include spheres, isotropic cylinders and ellipsoids, core–shell ellipsoids, and core–shell spheres.

Furthermore, two polymer chain models have been added: Kholodenko worm (Kholodenko, 1993 ▶) and Gaussian chain (Debye, 1947 ▶). For densely packed spheres, a model has been included based on the local monodisperse approximation (LMA), which is one of the few structure factors that can be directly implemented given the internal design of the MC method, coupled with the Percus–Yevick (PY) approximation (see §5) (Kinning & Thomas, 1984 ▶). This particular model combination will, hereafter, be referred to as ‘LMA-PY’.

*McSAS* can run with or without a user interface, enabling integration into existing data processing procedures. Multiple data files can be provided on the command line for batch fitting. The fitting procedure can then be automatically initiated, inheriting the settings of the previous GUI instance.

Graphical output and population statistics are calculated for a user-specified number of parameter ranges (regions of interest). The distributions can be shown with the (horizontal) parameter axes in logarithmic or linear scales, and the (vertical) amount number-weighted or volume-weighted. For broad distributions, however, volume-weighting is strongly recommended (see the note in §2.3.2). The distributions shown include the minimum observability limit, *i.e.* the minimum required amount for each contribution to be statistically significantly contributing.

Lastly, population statistics of the solution are determined independently of the histogramming procedure. For each selected parameter and range, the total value and the four distribution modes are provided: the mean, variance, skew and kurtosis. These are number- or volume-weighted depending on the user’s choice. Such population statistics simplify the analysis of population trends for *in situ* experiments or other inter-related data sets.

#### 2.3.2. Drawbacks   

Owing to its ‘brute force’, iterative nature, the method is not as fast as some of the alternatives mentioned in the *Introduction* [Sec sec1]. Optimization speed is strongly dependent on the accuracy of the data. A reasonably accurate data set (with small data uncertainties) may require a few minutes on a normal modern desktop computer. This is expected to improve in the near future through implementation of multithreading.

Secondly, there is a risk of under-specifying the fitting model when more complex models are chosen. For example, if a cylindrical scatterer model is chosen, and its length and radius are allowed to span the same size range, the solution is no longer unique and a multitude of valid solutions will be found. This manifests itself through excessive uncertainties in the result, originating from large discrepancies between the independent *McSAS* repetitions. Such ambiguity can be easily arrived at when using models such as core–shell objects and anisotropic objects. For these complex shapes, the allowed size ranges for the shape parameters may require the application of strict constraints before a unique solution is obtained.

Two common failure modes of the *McSAS* method can occur. The first happens when data are provided containing unrealistically low uncertainty estimates, which will lead to an attempt by *McSAS* to describe ostensibly significant data variations as features in the size distribution. This will lead either to a failure to reach convergence or to spurious features in the resulting parameter distribution. To alleviate this problem somewhat, the uncertainty is clipped to be at least 1% of the intensity value. Previously, this has been found to be a practical limit from data correction considerations (Pauw, Pedersen *et al.*, 2013 ▶) and is a value supported by experimental results (Hura *et al.*, 2000 ▶). This lower limit can be adapted or bypassed if better estimates can be guaranteed.

The second failure mode occurs when the fitting range is set too broad (beyond the range supported by the data). It is recommended to keep size parameters within the limits dictated by the *q* range of the data,^2^ estimated as 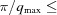 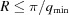. Exceeding these limits may result in spurious features appearing beyond these limits, as explored in the supplementary information given by Pauw, Pedersen *et al.* (2013 ▶).

Of further note is that small-angle scattering data represent a weighted distribution: volume-weighted according to Porod (Porod, 1952 ▶) or surface-weighted in terms of observability [explored, amongst others, in the article by Pauw, Pedersen *et al.* (2013 ▶)]. The implication of this is that volume-weighted size distributions can be easily retrieved using a Monte Carlo approach, as small to medium *volume fractions* of small-sized scatterers can be readily distinguished in scattering patterns. Not so for small *number fractions* of small-sized scatterers in a disperse mixture, for which exceedingly little evidence exists in most measured scattering patterns (and in particular for broad size distributions). Therefore, when (broad) size distributions determined using *McSAS* are shown in their number-weighted form, the values and uncertainties of the small-sized components can be seen to vary excessively (with some values becoming untenably large). Such issues are usually not encountered when using classical fitting methods.

Classical methods circumvent this issue, as the integral equation that is solved strictly constrains the distribution probability at all sizes (*e.g.* the probability distribution typically assumes a value of zero at its smallest size). These methods are, therefore, seemingly capable of determining even small number fractions of small scatterers accurately. However, the *evidence* for small numbers of small-sized scatterers may be very weak in the data. In summary, the strict assumptions placed in classical methods on the number-weighted size distribution shape may conceal the lack of evidence for the absence or presence of scatterers at the small end of the distribution, thus implying accuracy where there is none.

## 3. Current implementation   

### 3.1. User interface features   

**Figure 2:**
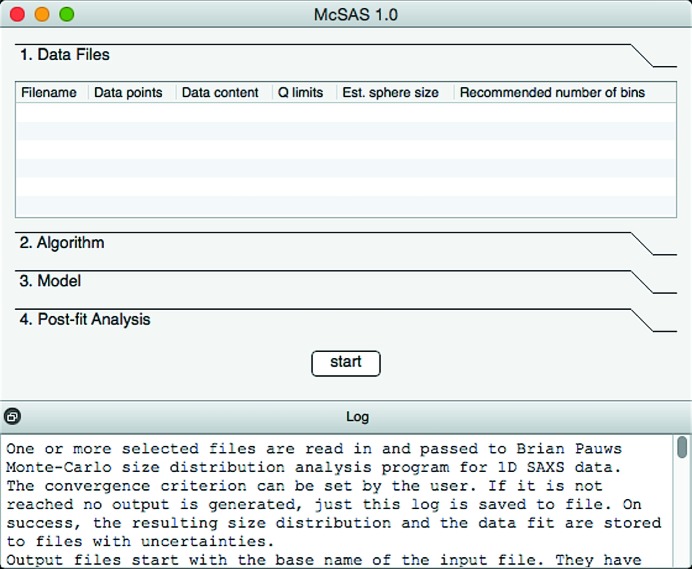
The main interface of the *McSAS* software upon startup, showing four configuration panels. The ‘Data Files’ panel allows selection and input of the data of interest, the ‘Algorithm’ panel contains settings to adjust the optimization method behaviour, ‘Model’ contains all parameters and settings relevant to the chosen morphology, and ‘Post-fit Analysis’ holds the settings for histogramming and visualization of the result.

The user interface is divided into several panels, each limited to a different aspect of the process (see Fig. 2 ▶). These consist of a ‘Data Files’ panel, an ‘Algorithm’ panel, a ‘Model’ panel and a ‘Post-fit Analysis’ panel, and will be discussed in order.

The ‘Data files’ panel shows data files loaded upon startup (as command-line arguments) or files added through the right-click menu. All files will be treated identically when the fit is run, though their order of processing can be changed as desired. Available data are read from the input file, which is expected to consist of three semicolon-separated columns of *q* (nm), *I* [(m sr)^−1^] and the uncertainty estimate 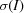 [(m sr)^−1^]. An optional fourth column can be used to indicate the azimuthal angle ϕ to aid fitting of anisotropic scattering patterns (a future feature). To help with determining reasonable limits of size parameters in particular, basic analysis is performed when loading each data file. The minimum and maximum values of the provided *q* vector are used to estimate the maximum and minimum possible scatterer size under the assumption of solid spherical scatterers. Those estimates are displayed next to each data file and, by double-click, can be applied as optimization limits for radius-type model parameters.

The ‘Algorithm’ panel contains a subset of MC algorithm settings addressable by the user (see Table 1 ▶). The most important of these is the chi-squared criterion. While this is per default set to 1, it may prevent reaching a state of convergence (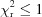) for data whose uncertainty estimates are insufficiently large or poorly estimated. Increasing this value will allow the convergence condition to be reached, after which the fit may be evaluated. This increase directly affects the uncertainties on the resultant distribution. Additionally, the number of shape contributions can be increased. While the default setting of 300 is sufficiently large to reach the convergence criterion for most scattering patterns, and small enough to reach it rapidly, there may be cases for which an increased number is desired. Using the timing information shown in the graphical output, the number of shape contributions can be optimized to reach convergence as fast as possible (a method discussed by Pauw, Pedersen *et al.* (2013 ▶). Likewise, the number of repetitions can be changed. These independent repetitions are used to estimate the uncertainties on the resultant size distribution, but a reduced number should suffice for initial testing. Lastly, a selection can be made on whether a flat background contribution is to be taken into account when matching the MC intensity to the detected signal.

The ‘Model’ panel contains all information on the model used to describe the scatterer morphology. The pulldown menu offers a selection of models that can be used to define the contributions’ scatterer shape. The associated parameters and options for the model chosen will then be shown on the right-hand side. Parameters which are selected for fitting require upper and lower bounds to be set.

The ‘Post-fit Analysis’ panel offers basic analysis capabilities for interpretation of the MC result. When a range entry is added, the user can select which parameter to histogram, what parameter range to consider (if they decide not to automatically follow the model parameter range) and how many bins to use. Increasing the number of histogram bins will lead to increased detail in the resulting histogram at the cost of larger uncertainties and evidence requirements *via* observability limits. Furthermore, a choice can be made whether to use a linear or logarithmic parameter scale (useful for distributions spanning several decades) and whether to plot volume- or number-weighted size distributions. When using absolute units, only the volume-weighted distribution will contain absolute values; the number-weighted distribution is normalized for lack of information.

Finally, the ‘Start’ button starts the process, and the ‘log’ shows the output of the program as it runs (and is automatically stored in a file).

### 3.2. Support, availability and licensing   

All *McSAS* information, including instructions and downloadable items, are available through http://mcsas.net/. A reasonable degree of support is provided by the authors subject to the availability of time. Instructional videos are available to help the user get started.

The software is written in the Python 2.7 programming language and available as a Git DVCSS repository. The interpreted code should run without issue on any desktop computer running any operating system that supports Python 2.7. It has been tested on the three main operating systems: Windows, Linux and Mac OS X. Standalone packages of stable versions are also available for these operating systems, which do not require Python to be available on the host computer. The software is released under an open-source GPLv3 license, allowing for academic and commercial adoption given proper attribution. Users for whom the software has been useful may refer to this work.

## 4. Application example 1: bimodal nanoparticle reference material   

To test the ability of the program to retrieve bimodal size distributions, a reference material was measured containing two fractions of silica nanoparticles [reference material ERM-FD-102 (Kestens & Roebben, 2014 ▶)]. The first fraction consists of 0.36 vol.% of 17 (2) nm-diameter nanoparticles. The second fraction consists of nanoparticles with a mean diameter of 89 (2) nm at a volume fraction of 0.018 vol.%. This makes the volume ratios of the two components 95 and 5%, respectively.

The measurements were performed on an Anton Paar slit-collimated instrument utilizing mirror-monochromated copper 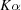 radiation and modified to use a Dectris Mythen detector. The data have been calibrated to absolute intensity using the methods described by Orthaber *et al.* (2000 ▶), the scaling of which was verified using a measurement of bovine serum albumin. The reference material, a water background and an empty capillary background were measured for 30 min each, corrected and desmeared using the software provided by Anton Paar. The data thus collected have been regrouped and averaged over 50 intervals, logarithmically spaced in *q*. The uncertainty has been set as the largest of (*a*) the propagated data uncertainty or (*b*) the standard error on the mean. None of these uncertainty estimates are smaller than 1% of the intensity value. The data span a *q* range of 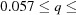 2.88 nm^−1^, corresponding to an estimated size range of 1.09 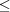 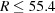
 nm.

**Figure 3:**
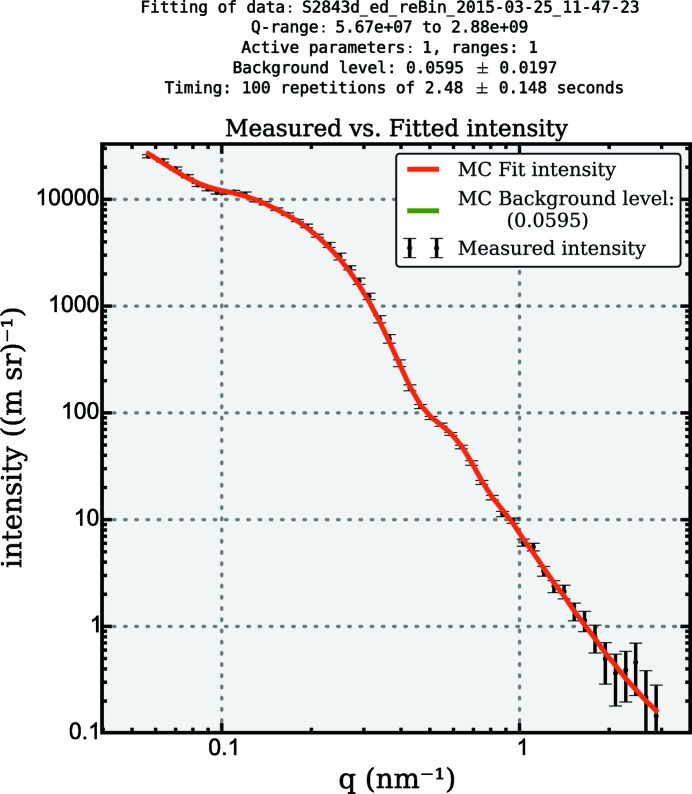
*McSAS* graphical output showing the best fit obtained using the MC method to a scattering pattern obtained from a mixture of dilute particles with certified diameter of 17 (2) nm and 89 (2) nm silica particles. The particle volume ratio of small to large particles is 19:1.

**Figure 4:**
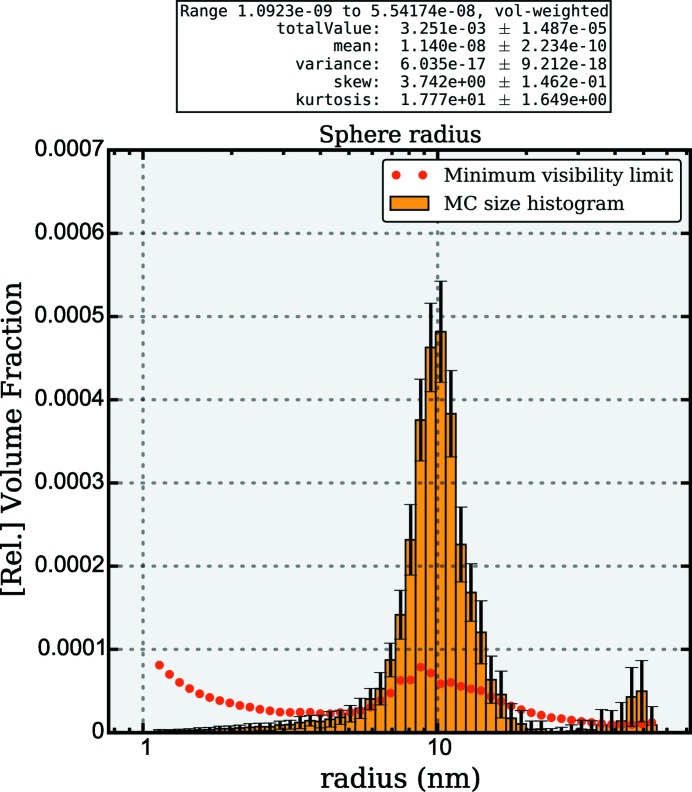
*McSAS* graphical output showing the volume-weighted size distribution associated with the MC fit of dilute silica particles shown in Fig. 3 ▶.

Analysis of the data using the *McSAS* program results in the fit shown in Fig. 3 ▶. To achieve this, the standard settings have been used, except for the number of repetitions (100) and the scattering length density difference (1.017
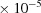
 Å^−2^). The two populations have been correctly resolved in the analysis (see Fig. 4 ▶), with the first population 1.09 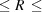 25 nm having a volume-weighted mean of 9.86 (6) nm and a volume fraction of 0.311 (3)%. The second population 25 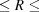 55.4 nm has a volume-weighted mean of 45 (2) and volume fraction of 0.013 (4)%.

Although the agreement is good, the values do deviate slightly from the stated values for the reference material. Furthermore, as the large-sized component is present at small volume fractions and close to the end of the measurement range, its characterization is not as accurate as that of the small component.

## 5. Application example 2: densely packed nanoparticles   

Dense systems add a degree of complexity to small-angle scattering and are, therefore, interesting as a test case for MC methods. A suitable data set of densely packed, dry SiO_2_ spheres (with a stated radius of 75 nm) has been provided by Peter Høghøj of Xenocs, as part of a demonstration data set measured on their Xeuss SAXS instrument. The SiO_2_ spheres are packed in a randomly jammed fashion, implying that the volume fraction *v*_f_ is approximately 0.63 (Song *et al.*, 2008 ▶).

**Figure 5:**
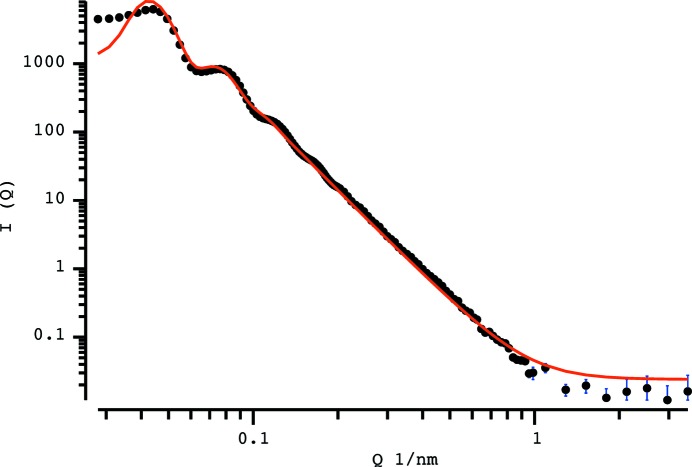
Best fit using a classical model (implemented in *SASfit*) to a scattering pattern obtained from packed silica spheres. Model uses a sphere form factor with a LMA-PY structure factor and a Gaussian size distribution.

A reasonable fit can be obtained using classical fitting methods implemented in *SASfit* (Bressler *et al.*, 2015 ▶), with a model of Gaussian distributed spheres and a structure factor consisting of a PY hard-sphere interaction model assuming the local monodisperse approximation, forming the aforementioned LMA-PY combination. This resulting fit is shown in Fig. 5 ▶. Most of the intensity can be described well, apart from the region at low *q*.

**Figure 6:**
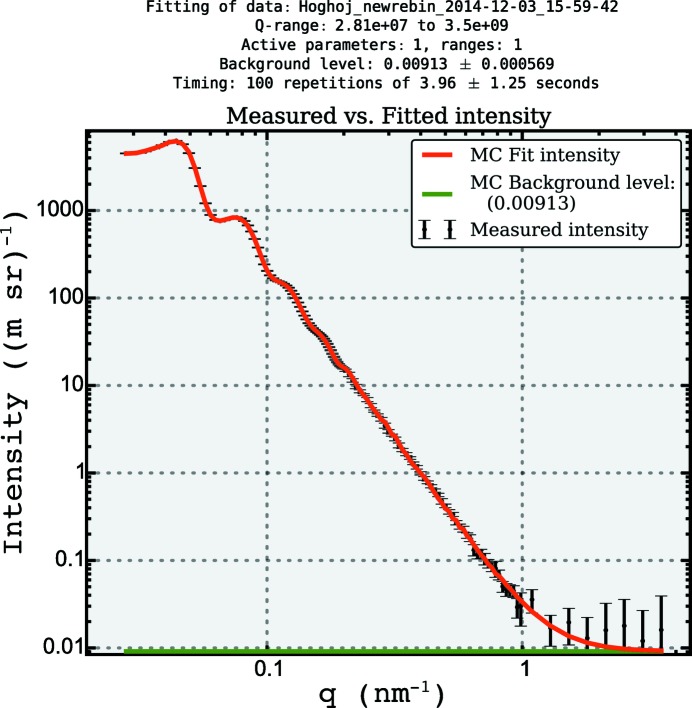
The best fit obtained using the MC method to a scattering pattern obtained from packed silica spheres. Model using a sphere form factor with a LMA-PY structure factor.

**Figure 7:**
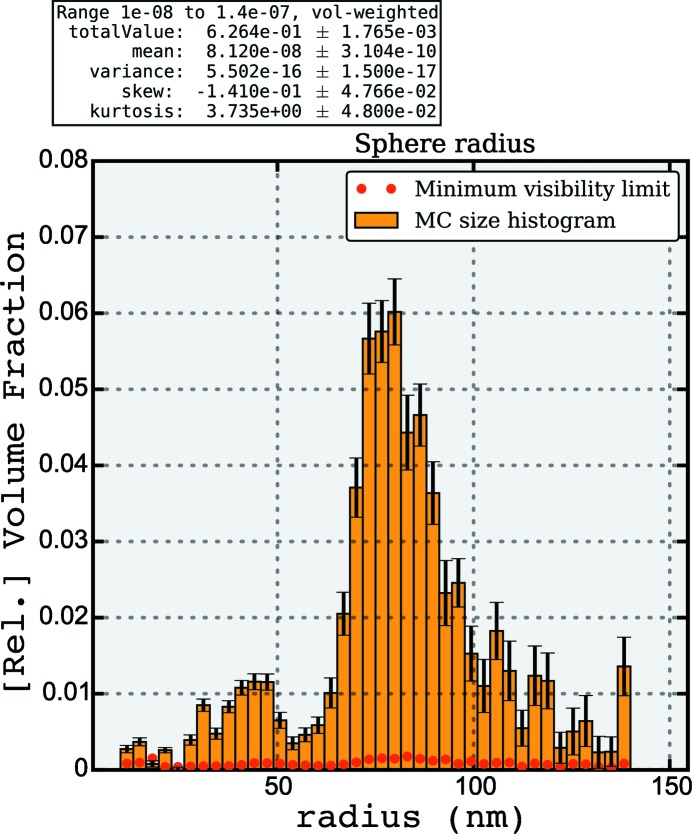
*McSAS* graphical output panel showing volume-weighted size distribution associated with the MC fit of Fig. 6 ▶.

A fit to within data uncertainty can be obtained using the same model in *McSAS* (see Fig. 6 ▶), with the volume fraction *v*_f_ set to 0.63. Note that the instrumental resolution has not been considered in either the classical or the MC approach. The main feature in the resultant size distribution (shown in Fig. 7 ▶) is indeed at the size indicated for the sample [with a number-weighted mean radius of 76.1 (2) nm], but a minor component is visible at about half the radius of the main component.

While the origin of the minor features cannot be established without further investigation, we have found that a similar good fit can be obtained when other volume fractions are set (possible between 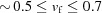). Changing the volume fraction drastically affects the size distribution and demonstrates that there are a multitude of solutions accessible through adjustment of the volume fraction. This highlights once more that information *must* be provided on the sample to allow SAS analyses to arrive at a unique solution. However, the overall result is quite satisfactory and a clear improvement compared with the classical approach.

## 6. Conclusion   

Although the *McSAS* package provides the user with a comprehensive model library, it can be readily extended with additional models. The relatively high computational effort increases with the complexity of new models, but should not be a major concern given the ever increasing availability of computing power. Concerning the significance of the resulting particle distributions, care must be taken that sufficient external information is provided to ensure a unique solution. This is of particular importance for complex models.

## Figures and Tables

**Table 1:** Selected program parameters and their effects on the computation For the advanced settings and defaults that can be found in the mcsasparameters.json file, only selected values are listed.

Location	Parameter name	Effect
GUI Algorithm panel	Convergence criterion	The least-squares value ( 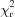 ) at which the fit is considered a success. For data with good uncertainty estimates, this can be set to 1. For a quick fit, it can be set to larger values. Values below 1 are not recommended.
	Number of repetitions	The number of independent optimizations to be run. Larger values will result in improved uncertainty estimates on the result (and a slightly smoother result), but calculation time increases proportionally.
	Number of contributions	The number of individual contributions whose weighted sum comprises the total model intensity. Too few or too many will result in slow optimization times. Most patterns can be fitted using 300 contributions quickly, but times can be optimized using the timing information shown in the result.
	Find background level	If selected, a flat background is fitted during matching of model and data. This speeds up the fit with minimal effect on the result, as many scattering patterns contain a flat scattering component as well (due to density variations or incoherent scattering).

GUI Post-fit Analysis panel	Parameter	The parameter to show the distribution of.
	Lower upper	The distribution will be shown in this parameter range only. This can be used to cut off regions outside the range of interest. Population statistics also apply only to this range.
	Number of bins	The number of divisions to use in the distribution display. By increasing this number, more detail *may* be visible provided one stays within the Shannon channel limit (indicated in the ‘Data Files’ panel). An increase in the number of divisions will also negatively affect uncertainty estimates and observability limits.
	*X*-axis scaling	Scaling (linear or logarithmic) for the parameter axis of the distribution. Logarithmic recommended for wide parameter ranges.
	*Y*-axis weighting	The vertical axis can be shown in volume or number distributions. Volume-weighted distributions recommended; number-weighted distributions can be used for samples with a narrow dispersity.

mcsasparameters.json (file, advanced settings and defaults)	maxIterations	If convergence has not been reached within this number of iterations, the optimization attempt is aborted. Larger values may allow complex calculations to finish successfully, but often nonconvergence can be traced back to poor initialization settings. Increasing this value increases the maximum possible calculation time.
	compensationExponent	Adjusts internal weighting of scattering pattern contributions. Adjustment between 0.3 and 0.7 may lead to slight speed increases for some samples.
	eMin	Minimum uncertainty estimate in fraction of intensity. Default 0.01 sets the uncertainty value to be no less than 1% of the data intensity value. Can be increased or reduced based on best guess estimate for minimum inter-related data point uncertainty. A too low value may prevent reaching convergence.

## Footnotes

1 The scattering patterns of the individual contributions of the set are weighted with the inverse of their volume to obtain a more evenly matched impact on the total (Porod, 1952 ▶). This process drastically reduces the required number of contributions and improves fitting flexibility. For most practical scattering patterns we encountered, about 200–300 contributions are sufficient to describe the scattering patterns with a minimum of optimization iterations.

2 More precisely, it is the *q* point spacing which defines the upper limit. However, there is a tendency during the azimuthal averaging procedure to over-specify the number of bins, leading to excessively small *q* point spacing.
